# The development and acceptability testing of an app-based smart survey system to record smoking behaviour, use of nicotine replacement therapy (NRT) and e-cigarettes

**DOI:** 10.1186/s13104-022-05983-8

**Published:** 2022-03-10

**Authors:** Yue Huang, Joanne Emery, Felix Naughton, Sue Cooper, Lisa McDaid, Anne Dickinson, Miranda Clark, Darren Kinahan-Goodwin, Ross Thomson, Lucy Phillips, Sarah Lewis, Sophie Orton, Tim Coleman

**Affiliations:** 1 Division of Epidemiology and Public Health, University of Nottingham, Nottingham, NG7 2RD UK; 2 Division of Primary Care, University of Nottingham, Nottingham, NG7 2RD UK; 3 School of Health Sciences, University of East Anglia, Norwich, NR4 7UL UK

**Keywords:** Survey, Smoking, Pregnancy, Nicotine replacement therapy, e-cigarettes, Smartphone app, Medication adherence, Ecological momentary assessment

## Abstract

**Objective:**

Nicotine replacement therapy (NRT) helps people stop smoking. Monitoring treatment adherence is important as poor adherence to NRT limits its effectiveness. As e-cigarettes contain nicotine, their use (‘vaping’) is likely to affect both NRT use and smoking. We wished to measure adherence to NRT, and to investigate relationships between NRT, vaping and smoking so we developed ‘NicUse’, a smartphone App linked to a cloud database for collecting data relevant to NRT adherence. We report user-acceptability and investigate data validity among pregnant people by comparing heaviness of smoking reported to NicUse surveys with contemporaneous exhaled carbon monoxide readings.

**Results:**

Thirty five pregnant women participating in a pilot study were asked to install and use NicUse on their smartphones. 32/35 (91%) logged into NicUse, 31 (89%) completed one or more surveys, and 22 (63%) completed these on ≥ 20 of 28 study days. Twenty-four gave end-of-study user acceptability ratings; 23 (96%) agreed or strongly agreed NicUse was ‘Easy to use’ and ‘Instructions were clear’. There was a strong correlation between the number of daily cigarettes reported on NicUse and exhaled CO readings taken on study Day 7 (Pearson’s r = 0.95, p < 0.001). NicUse appears highly acceptable, and smoking data reported to it shows validity.

**Supplementary Information:**

The online version contains supplementary material available at 10.1186/s13104-022-05983-8.

## Introduction

Smoking in pregnancy increases risks of miscarriage, premature birth, stillbirth, low birth weight and sudden unexpected death in infancy [1]. In 2020–2021, 9.5% of mothers in England smoked [2], and reducing this to 6% by 2023 is an ambition of the 2017 Tobacco Control Plan for England [3]. Nicotine replacement therapy (NRT) helps non-pregnant people stop smoking [4] but seems less effective in pregnancy [5], in part because pregnant women’s adherence to NRT is typically poor and they tend not to use high enough doses for sufficiently long [6]. A method of accurately assessing pregnant women's adherence to NRT alongside concurrent nicotine e-cigarette use and smoking is needed in order to evaluate interventions that aim to increase adherence and reduce smoking, but there are no standard approaches for doing this. Previous studies have used retrospective surveys and interviews and ‘pill counts’ to assess medication adherence [7], but these approaches can create bias and have practical limitations. We therefore developed NicUse, a smartphone app combined with a ‘cloud’ database technology, to obtain longitudinal data on these outcomes. This study investigated the acceptability and validity of using NicUse for the daily collection of research data on smoking, NRT and e-cigarette use.

## Main text

### The smartphone survey approach

Surveys are a quantitative research data collection method used for describing or comparing knowledge and attitudes to phenomena and behaviours. For example, to assess adherence with drug treatments, surveys may collect information about drug products or brands and doses used [8]. The use of surveys in research is to ask pre-planned questions to collate specific data [9] but taking surveys can be time-consuming and costly. Recently, smart mobile devices have been used to collect research information via questionnaires [10–12], and smartphone apps can obtain high-quality data [10]. There are limitations with traditional data collection methods for robustly collecting NRT adherence data, including retrospective recall bias risk due to the typical elapsed time between the event and when data is collected, sub-optimal response rates, cost and participant burden. We developed an app (NicUse) to administer simple, daily surveys on smoking and NRT/e-cigarette use. We linked app surveys to a cloud database (see below) to enable continuous monitoring of survey responses, and send prompts to non-respondents.

### The Cloud

Cloud computing is the online delivery of computing services like software, databases, servers, and networking which users can access from locations with internet connections. Researchers using Cloud technology can focus on optimising methods for collecting and transferring data to Cloud databases as maintenance, functionality and security of online services are dealt with by the Cloud provider.

### NicUse

**Fig. 1:**
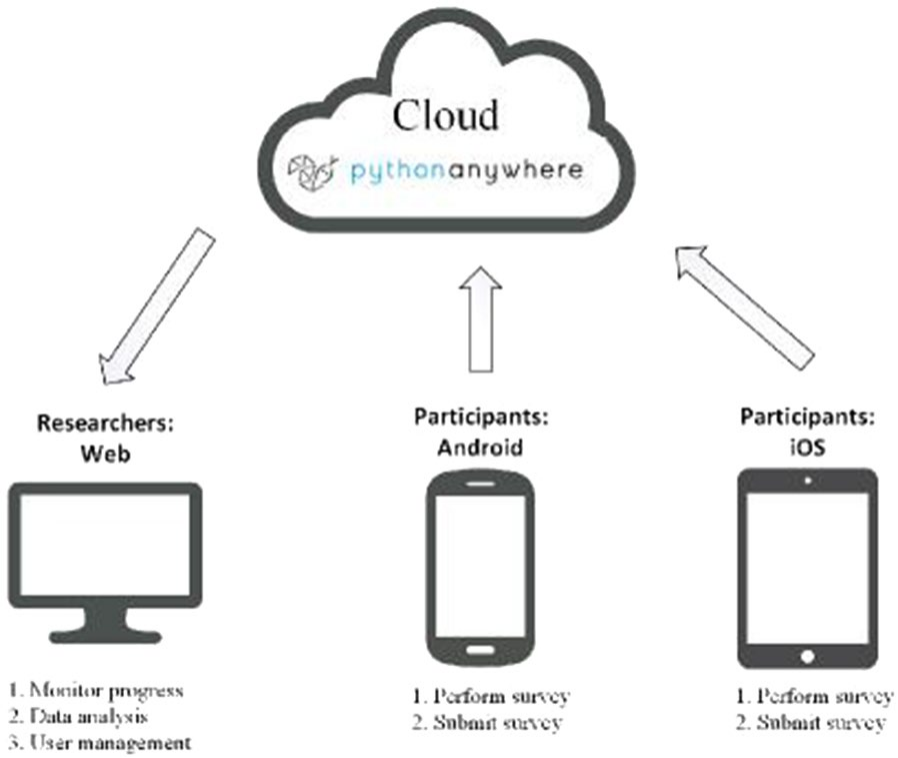
NicUse system

The NicUse App comprises a smartphone user interface and a web-portal, which facilitates researchers’ access to survey data. Figure 1 illustrates key NicUse system components. NicUse is available in Google Play (Android) and App Stores (iOS). The Cloud used by this app is based on an Amazon web service through Pythonanywhere as PaaS (Platform as a Service).

#### NicUse participant interface

**Fig. 2:**
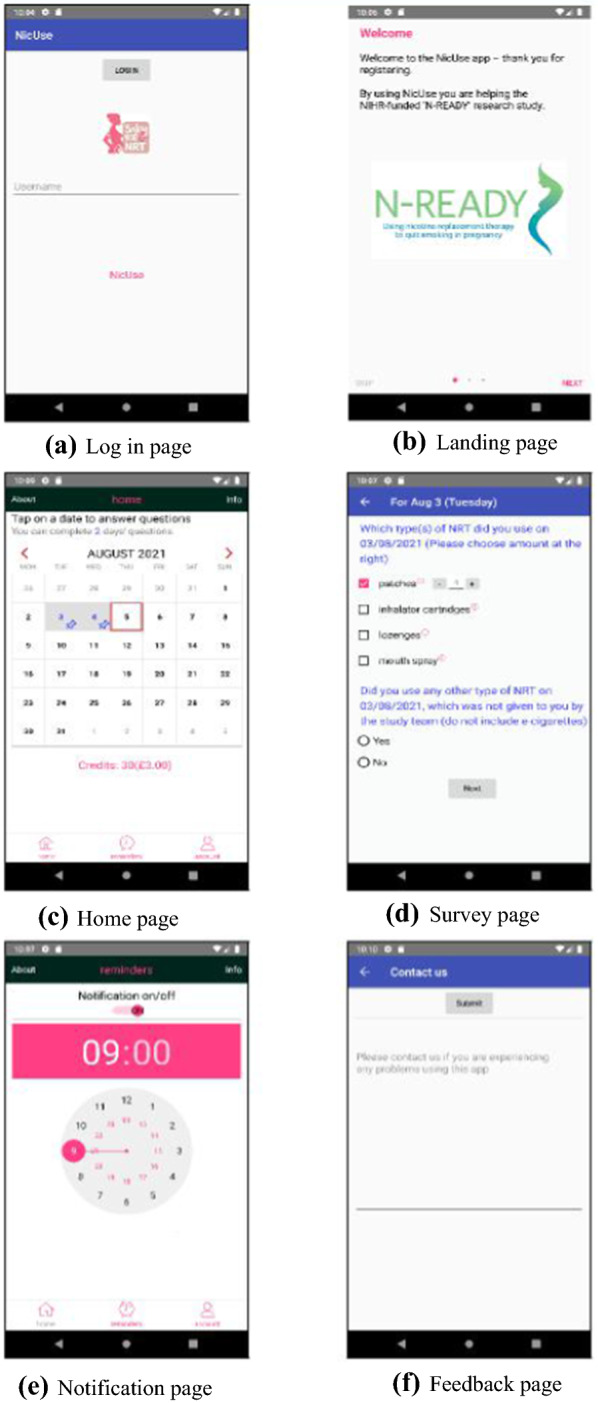
User Interface of NicUse

Figure 2 demonstrates how NicUse looks on Android devices and it looks similar on iOS devices. Users are assigned a unique code for app access. Figure 2a shows the log in page, and Fig. 2b is the landing page seen after logging in. The app home page (Fig. 2c) includes a calendar, on which a pink tick below a date indicates a survey for that day has been completed, but a blue pin indicates survey non-completion. Users tap ‘blue pin’ dates to start surveys. To minimise recall error, participants can only complete surveys for the previous two days ago. The top of the home page shows the total number of surveys left to complete. Figure 2d demonstrates one of the survey pages displayed after tapping on a ‘blue pin’ date; this page asks about NRT use. Users are always routed through the survey regardless of their response; each page provides ‘pop-up’ instructions explaining how to answer questions, and alerts appear if potentially implausible responses are entered. Before surveys can be submitted, users confirm answers or edit incorrect responses, and after submission data are immediately transferred to a Cloud database rather than being stored on their smartphone.

Credits are awarded for survey completion (equivalent to 50p per day) and a running total of credits earned is displayed below the summary page calendar; after submitting surveys users are notified how many new credits they have earned. Daily survey completion can be incentivised by linking virtual credits to real rewards (e.g. gift cards). Participants can set up daily app completion reminders (Fig. 2e) and send feedback to the study team via the app (Fig. 2f).

#### NicUse researcher interface

**Fig. 3:**
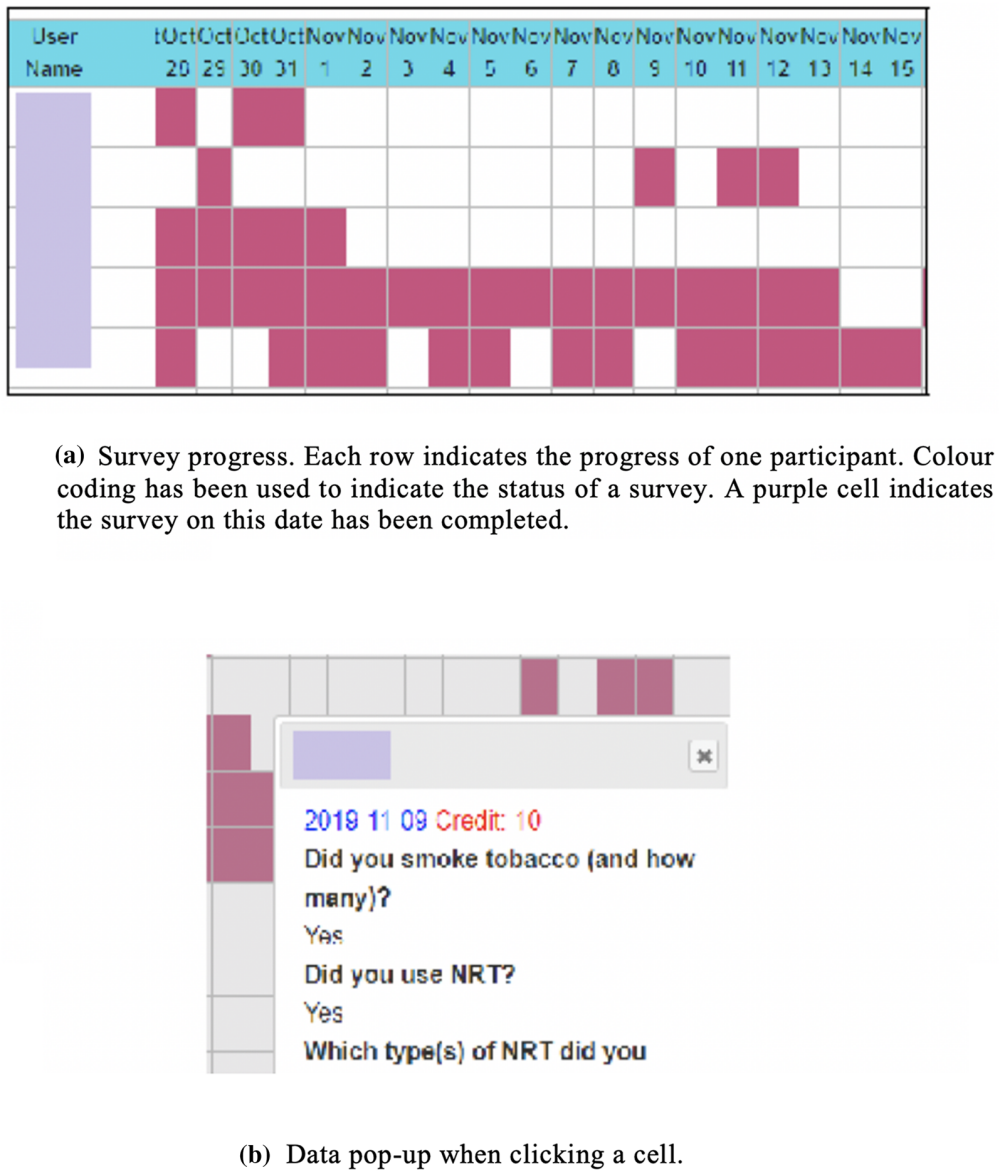
NicUse Web portal for researchers

Figure 3 shows the web portal researcher interface. This can be used for monitoring survey completion, managing participants’ accounts, online data analysis, troubleshooting, and collation of user feedback. Monitoring of survey completion is illustrated in Fig. 3a; cells represent days and turn from white to purple when the survey is submitted. When a purple cell is double-clicked, the survey data appear in a ‘pop-up’ (Fig. 3b). This interface facilitates proactive survey management as researchers or clinicians can identify and, if desired, contact participants who have not completed surveys. Similarly, the portal makes it possible to identify participants who give particular answers to questionnaire item(s) whilst research is ongoing. Via this interface, researchers can set surveys to begin from specific dates (e.g. the smoking quit date of a study participant). Data can also be downloaded from the web portal in MS Excel format whenever required.

### NicUse beta-testing

Following initial testing within the research team, beta testing involved 14 non-pregnant women known to researchers. Some were members of our Patient and Participant Involvement (PPI) panel, with previous experience of smoking and/or NRT use in pregnancy, and others had experience of developing/evaluating behavioural apps. Nine had Android phones and five had iPhones. Women used the NicUse system for at least 1 week, inputting fictional smoking, e-cigarette and NRT data then providing feedback by email or telephone. Feedback resulted in minor cosmetic and wording changes. Women were compensated for their time with £20 in shopping gift cards.

### User experience of NicUse data collection (acceptability testing)

As part of a research programme aiming to optimise an intervention for improving pregnant women’s adherence to NRT, we recruited three sequential cohorts of pregnant women who agreed to try stopping smoking with NRT (total N = 40). Participants were asked to download NicUse and to report their daily smoking, NRT and e-cigarette use on the app for each of the 28 days following their agreed date for quitting smoking. Between cohorts we made very minor changes to survey items; for example, in Cohort 1 we simply asked for smoking status (yes/no) whereas, in Cohorts 2 and 3, we also sought the number of cigarettes smoked each day. Five of 20 participants in Cohort 3 withdrew before any stop smoking support was provided or NRT offered, and so did not have the opportunity to log in to or use NicUse. Of the 35 participants who used support and NRT, 32 (91%) logged in to the app, 31 (89%) submitted data at least once between their quit date and day 28 (7/8 in Cohort 1; 12/12 in Cohort 2; 12/15 in Cohort 3) and 22/35 (63%) reported data on at least 22 out of 28 days. An additional data file with table and bar chart shows the results in more detail (see Additional file 1). Women who did not submit NicUse surveys also failed to respond to other data collection requests (e.g. end-of-study evaluation), indicating disengagement/discontinuation from the study.

Among the 24 participants who responded to the end-of-study evaluation at 28-day follow up, NicUse user acceptability ratings were high. On a 5-point rating scale from ‘Strongly Disagree’ to ‘Strongly Agree’, 23 (96%) either strongly agreed or agreed that NicUse was ‘Easy to use’ and that ‘Instructions were clear’, and 21 (88%) strongly agreed or agreed that it was convenient to report NRT or e-cig use or smoking behaviour using the app. Concerns about privacy of data submitted were low: 19 (79%) participants strongly disagreed or disagreed that they had any such concerns. Twenty participants (83%) agreed that they found it easy to remember to report on the app and twenty-one (87%) agreed that they found it easy to remember exact amounts of NRT used. Twelve (50%) strongly agreed or agreed that credits and rewards helped increase reporting frequency and 21 (88%) found in-app reminders helpful.

Qualitative end-of-study interviews with N = 25 cohort participants supported the ease of NicUse e.g. “I found it very simple, easy, the questions weren’t confusing or anything” (Participant 02, site 2, Cohort 2). Two participants suggested allowing completion of surveys from more than two days earlier, but they also felt that accuracy of recall might be compromised; in general, women preferred to report daily as this “turned [survey completion] into a habit” (Participant 01, site 1, Cohort 3), and with daily reporting, recall of NRT use was considered both easier and more accurate by participants.

### Exploration of NicUse data validity

Irrespective of whether they were smoking or not, cohort study participants were asked to provide exhaled air carbon monoxide (CO) readings on Day 7 following their quit date. Seventeen provided CO readings, and 11 of these women were in Cohorts 2 and 3 where NicUse sought the number of cigarettes smoked each day. The median (range) of exhaled CO readings provided was 2 ppm (0–10ppm) and the mean (SD), 4.1 (5.4). Ten of these 11 women also recorded the number of cigarettes smoked per day (CPD) on the NicUse survey on the same day as the exhaled air CO sample; the CPD median (range) was 0 (0–10) cigarettes and the mean (SD), 1.6 (3.2). CO readings and reported CPD were highly correlated (Pearson’s r = 0.95, p < 0.001).

### Conclusions

Most women logged into NicUse and feedback was overwhelmingly positive, so we believe the app is acceptable for research and clinical use. As pregnant women who smoke are often disadvantaged and can be very hard-to-reach, we feel optimistic about using NicUse in other smoking populations. The strong correlation between heaviness of smoking reported to NicUse and exhaled CO readings suggests NicUse provides valid data on tobacco smoke exposure. In summary, the NicUse system appears feasible to use for measuring adherence to NRT, smoking behaviour and e-cigarette use and merits further evaluation.

## Limitations

NicUse may be restricted to use in more technologically-developed or high-income economies because respondents need regular access to smartphones, and internet, mobile data signals. Even within high income, technologically-developed countries, this kind of data collection may not be appropriate within the most deprived and elderly (non-pregnant) communities who may not have ready access to the internet or sufficient familiarity with smartphones [14, 15]. In addition, self-reporting of data can involve errors. For example, a NicUse user who had smoked a few puffs of a cigarette might not believe this was sufficient to warrant a report of having smoked. We tried to avoid this by providing ‘pop ups’ guidance alongside app survey items. However, apart from returning used NRT packaging, all practical methods for measuring adherence to NRT rely on self-report, and the validity of NicUse smoking data indicates that reported NRT data are valid.

It is possible that lower engagement with NicUse would have occurred if we had not offered financial incentives for data completion; however, these were small and commensurate with the amount that a study participant would typically be reimbursed for their time. In end-of-study interviews, some cohort participants had forgotten about the incentives and half of those present at follow up agreed that these did not help to increase their frequency of reporting. A final limitation is that feedback was not available from the five participants who were recruited to cohort studies but did not download the app and did not engage with the intervention or other study procedures; one possible explanation for this is that participants were deterred from using the app due to the requirement to complete daily surveys. However, we cannot be sure that this was the case and in all research studies, some participants will avoid engaging with study interventions.

## Supplementary Information

**Additional file 1: Table S1.** Number of days (out of 28) a NicUse report was submitted by participants.

## Data Availability

The data used to support the results were generated during the study, and has been submitted with the paper in “Additional file 1.”.

## Abbreviations

NRT: Nicotine replacement therapy
PPI: Patient and participant involvement
CO: Carbon monoxide
SD: Standard deviation
CPD: Cigarettes smoked per day

## References

[1] Cooper S, Orton S, Leonardi-Bee J (2017). Smoking and quit attempts during pregnancy and postpartum: a longitudinal UK cohort. BMJ Open.

[2] Statistics on Women's Smoking Status at Time of Delivery:England-Quarter4,2020–21, https://digital.nhs.uk/data-and-information/publications/statistical/statistics-on-women-s-smoking-status-at-time-of-delivery-england/statistics-on-womens-smoking-status-at-time-of-delivery-england---quarter-4-2020-21. Accessed 9 Aug 2021.

[3] Towards a smoke free generation—a tobacco control plan for England. https://assets.publishing.service.gov.uk/government/uploads/system/uploads/attachment_data/file/630217/Towards_a_Smoke_free_Generation_-_A_Tobacco_Control_Plan_for_England_2017-2022__2_.pdf. Accessed 9 Aug 2021.

[4] Hartmann‐Boyce J, Chepkin SC, Ye W, Bullen C, Lancaster T. Nicotine replacement therapy versus control for smoking cessation. Cochrane Database Syst Rev. 2018;5. 10.1002/14651858.CD000146.pub5. 10.1002/14651858.CD000146.pub5 PMC6353172 29852054

[5] Bowker K, Lewis S, Coleman T, Cooper S (2015). Changes in the rate of nicotine metabolism across pregnancy: a longitudinal study. Addiction.

[6] Claire R, Chamberlain C, Davey MA, Cooper SE, Berlin I, Leonardi‐Bee J, Coleman T. Pharmacological interventions for promoting smoking cessation during pregnancy. Cochrane Database Syst Rev. 2020;3. 10.1002/14651858.CD010078.pub3. 10.1002/14651858.CD010078.pub3 PMC7059898 32129504

[7] Hollands GJ, Naughton F, Farley A, Lindson N, Aveyard P. Interventions to increase adherence to medications for tobacco dependence. Cochrane Database Syst Rev. 2019;8. 10.1002/14651858.CD009164.pub3. 10.1002/14651858.CD009164.pub3 PMC6699660 31425618

[8] Alam I, Khusro S, Rauf A, Zaman Q. Conducting surveys and data collection: from traditional to mobile and sms-based surveys. Pak J Stat Oper Res. 2014;169–187. 10.18187/pjsor.v10i2.758.

[9] Pfleeger SL, Kitchenham BA (2001). Principles of survey research: part 1: turning lemons into lemonade. ACM SIGSOFT Softw Eng Notes.

[10] Miller Y, DiCiccio C, Lavista J, Gore-Felton C, Acle C, Hancock J, Richardson A, Nelson L, Palesh O, Oakley-Girvan I (2018). Smart (phone) approaches to mobile app data collection. Surv Pract.

[11] Mollard E, Michaud K (2018). A mobile app with optical imaging for the self-management of hand rheumatoid arthritis: pilot study. JMIR Mhealth Uhealth.

[12] Early J, Gonzalez C, Gordon-Dseagu V, Robles-Calderon L (2019). Use of mobile health (mhealth) technologies and interventions among community health workers globally: a scoping review. Health Promot Pract.

[13] Fischer F, Kleen S (2021). Possibilities, problems, and perspectives of data collection by mobile apps in longitudinal epidemiological studies: scoping review. J Med Internet Res.

[14] Kearns A, Whitley E (2019). Associations of internet access with social integration, wellbeing and physical activity among adults in deprived communities: evidence from a household survey. BMC Public Health.

[15] Digital divide persists even as Americans with lower incomes make gains in tech adoption, https://www.pewresearch.org/fact-tank/2021/06/22/digital-divide-persists-even-as-americans-with-lower-incomes-make-gains-in-tech-adoption/. Accessed 9 Aug 2021.

