# Supplementary material for: The development and acceptability testing of an app-based smart survey system to record smoking behaviour, use of nicotine replacement therapy (NRT) and e-cigarettes
Source: BMC Res Notes. 2022 Mar 10;15:100. doi: 10.1186/s13104-022-05983-8 (PMC8908557; doi:10.1186/s13104-022-05983-8)

| **Table 1: Number of days (out of 28) a NicUse report was submitted by participants** | | | |
| --- | --- | --- | --- |
|  | | Frequency | Percent |
|  | 0 | 4 | 11.4 |
|  | 3 | 2 | 5.7 |
|  | 4 | 1 | 2.9 |
|  | 5 | 1 | 2.9 |
|  | 6 | 2 | 5.7 |
|  | 7 | 1 | 2.9 |
|  | 16 | 1 | 2.9 |
|  | 19 | 1 | 2.9 |
|  | 22 | 3 | 8.6 |
|  | 23 | 1 | 2.9 |
|  | 24 | 1 | 2.9 |
|  | 25 | 5 | 14.3 |
|  | 26 | 1 | 2.9 |
|  | 27 | 4 | 11.4 |
|  | 28 | 7 | 20.0 |
|  | Total | 35 | 100.0 |


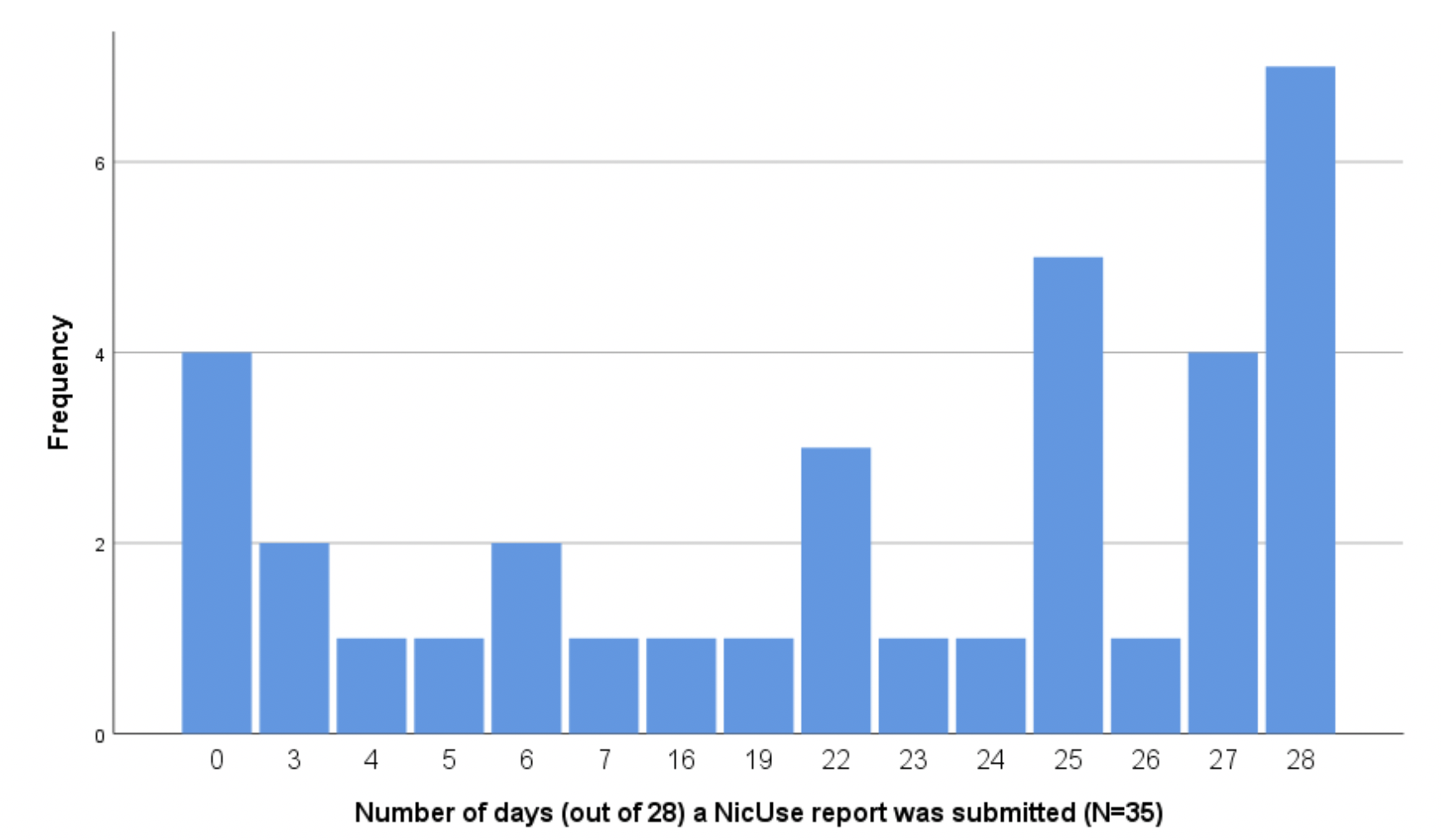

Supplement: Supplementary file 1 — Additional file 1: Table S1. Number of days (out of 28) a NicUse report was submitted by participants. [file 13104_2022_5983_MOESM1_ESM.docx]
